# Wernicke’s functional neuroanatomy model of language turns 150: what became of its psychological reflex arcs?

**DOI:** 10.1007/s00429-024-02785-5

**Published:** 2024-04-06

**Authors:** Ardi Roelofs

**Affiliations:** Donders Institute for Brain, Cognition and Behaviour, Centre for Cognition, Radboud University, Thomas van Aquinostraat 4, 6525 GD Nijmegen, The Netherlands

**Keywords:** Aphasia, Naming, Repetition, Tractography, Wernicke

## Abstract

Wernicke (Der aphasische Symptomencomplex: Eine psychologische Studie auf anatomischer Basis. Cohn und Weigert, Breslau. https://wellcomecollection.org/works/dwv5w9rw, 1874) proposed a model of the functional neuroanatomy of spoken word repetition, production, and comprehension. At the heart of this epoch-making model are psychological reflex arcs underpinned by fiber tracts connecting sensory to motor areas. Here, I evaluate the central assumption of psychological reflex arcs in light of what we have learned about language in the brain during the past 150 years. I first describe Wernicke’s 1874 model and the evidence he presented for it. Next, I discuss his updates of the model published in 1886 and posthumously in 1906. Although the model had an enormous immediate impact, it lost influence after the First World War. Unresolved issues included the anatomical underpinnings of the psychological reflex arcs, the role of auditory images in word production, and the sufficiency of psychological reflex arcs, which was questioned by Wundt (Grundzüge der physiologischen Psychologie. Engelmann, Leipzig. http://vlp.mpiwg-berlin.mpg.de/references?id=lit46, 1874; Grundzüge der physiologischen Psychologie (Vol. 1, 5th ed.). Engelmann, Leipzig. http://vlp.mpiwg-berlin.mpg.de/references?id=lit806, 1902). After a long dormant period, Wernicke’s model was revived by Geschwind (Science 170:940–944. 10.1126/science.170.3961.940, 1970; Selected papers on language and the brain. Reidel, Dordrecht, 1974), who proposed a version of it that differed in several important respects from Wernicke’s original. Finally, I describe how new evidence from modern research has led to a novel view on language in the brain, supplementing contemporary equivalents of psychological reflex arcs by other mechanisms such as attentional control and assuming different neuroanatomical underpinnings. In support of this novel view, I report new analyses of patient data and computer simulations using the WEAVER++/ARC model (Roelofs 2014, 2022) that incorporates attentional control and integrates the new evidence.

## Introduction

One hundred fifty years ago, 26-year-old Carl Wernicke published a monograph entitled *Der aphasische Symptomencomplex: Eine psychologische Studie auf anatomischer Basis* (The aphasic symptom complex: A psychological study on anatomical basis). The book described a model for the functional neuroanatomy of word repetition, production, and comprehension. At the heart of the model are psychological reflex arcs underpinned by fiber tracts connecting sensory to motor areas of the brain. According to Wernicke (1874), word repetition is achieved by fibers that associate the auditory image of a word in left superior temporal gyrus (STG) with its movement image in left inferior frontal gyrus (IFG) via the insula; concept-driven word production is achieved by fibers, including the arcuate fasciculus (AF), associating visual, auditory, tactile, olfactory, and taste images in distinct posterior cortical areas, making up a concept, with the movement image in the IFG; and word comprehension is achieved by fibers associating the auditory image of the word with the sensory images making up the corresponding concept. This epoch-making model has guided many research efforts and clinical applications. To mark the 150th publication anniversary of Wernicke’s book, I evaluate its central assumption of psychological reflex arcs in light of what we have learned about language in the brain since the book appeared.

The remainder is organized as follows. First, I describe Wernicke’s 1874 model and the evidence he presented for it. Next, I discuss his further clarification of the model (Wernicke 1886) and his final update, which was posthumously published (Wernicke 1906). Although the model had an enormous immediate impact, it lost influence after the First World War. Unresolved issues included the anatomical underpinnings of the psychological reflex arcs (e.g., Dejerine 1895; von Monakow 1897), the role of auditory images in word production (e.g., Lichtheim 1885a, b), and the sufficiency of psychological reflex arcs, which was questioned by Wundt (1874, 1902). After a long dormant period, Wernicke’s model was revived by Geschwind (1970, 1974). Although known in the literature as the “Wernicke-Geschwind” model (e.g., Anderson et al. 1999) or the “Wernicke-Lichtheim-Geschwind” model (e.g., Tremblay and Dick 2016), the version of Geschwind differed in several important respects from Wernicke’s original. Finally, I describe how modern research has led to a novel view of language in the brain, complementing contemporary equivalents of psychological reflex arcs with other mechanisms such as attentional control and assuming different neuroanatomical underpinnings. I report new analyses of patient data and computer simulations using the WEAVER++/ARC model (Roelofs 2014, 2022) supporting the novel view. This model was chosen for this article because it incorporates attentional control and integrates the new evidence.

## Wernicke’s model and its support

Wernicke published his 1874 monograph while he was an assistant physician at the Allerheiligen Hospital (All Saints Hospital) in Breslau. The monograph was inspired by a stay of six months with Theodor Meynert in Vienna, where Wernicke had learned about Meynert’s new neuroanatomical work. Shortly after publishing his monograph at the end of 1874, Wernicke went to Berlin to become an assistant to Carl Westphal and later founded a private clinic there. In 1885, he succeeded his former mentor Heinrich Neumann as professor of psychiatry in Breslau. After several years of problems with the Breslau municipal administrators, who even prohibited Wernicke to demonstrate patients during his lectures, he left for Halle. In 1905, Wernicke was killed in a bicycle accident, leaving behind a young wife and three children. Wernicke had several assistants and students who would later make their own important scientific contributions, including Heinrich Lissauer, Otfrid Foerster, Heinrich Sachs, Hugo Liepmann, Karl Bonhoeffer, Kurt Goldstein, Karl Heilbronner, and Karl Kleist. Geschwind (1967, 1974) described Wernicke’s work and his Breslau School, and its impact on the history of aphasia. For a biography of Wernicke and English translations of several of his works, including Wernicke (1886, 1893, 1906), see Eggert (1977).

In part I (pp. 3–12) of the 1874 monograph, Wernicke outlined a general theory of neuroanatomically grounded psychological reflexes, which he attributed to Meynert. In part II (pp. 12–38), he described his functional neuroanatomy model of language, formalized as diagrams, and applied it to aphasic word production, comprehension, and repetition. In part III (pp. 38–70), Wernicke discussed the aphasic symptoms of ten patients, four with autopsy results, which were taken to support his theory. He acknowledged that his views on aphasia were not new: “I am far from thinking that in the foregoing I have expressed entirely new views on the nature of aphasia. … What differs in my view from the earlier ones, however, is that the anatomical basis has been retained throughout”^1^ (pp. 67–68). Therefore, I restrict my discussion to patients with autopsy. These patients had been clinically examined by Wernicke in October and November 1873 (Case 2, Susanne Rother) and between December 1873 and May 1874 (all other cases), and three of them died before he finished his manuscript of the monograph in May 1874. In an addendum (pp. 71–72), he described the autopsy results of one more of the patients (his Case 8, Louise Funke), who had died in June 1874.

### The psychological model on anatomical basis of 1874

At the heart of Wernicke’s model are psychological reflex arcs that map sensory images in posterior brain areas onto movement images in anterior areas:

It suffices ... to explain the spontaneous movement in the manner of a reflex process. Anatomical pathways, which may mediate such psychological reflex actions, exist in abundance; the greater part of the cerebral white matter consists of such bundles of associations, some of which are simple, some of which are more complicated. (Wernicke 1874, pp. 10–11)

According to Wernicke (1874), the only distinction between a reflex movement and a psychological reflex movement, which underlies voluntary action, is that while the former is innate, in the latter there is a learned association between a sensory image and an image of a preformed movement, both of which are memory images (see also Kussmaul 1877; Lichtheim 1885a, b). While a reflex movement immediately follows stimulation, a psychological reflex movement follows the activation of a sensory image.^2^ Wernicke assumed that when multiple movement images are activated, the actual movement is determined by the image with the most or strongest associations or associated with the most intense sensory images. He stated: “The only right scientific definition of free will is in perfect agreement with this mechanical view of the origin of spontaneous movement” (p. 12). This makes it clear that Wernicke did not assume another factor, such as an influence of will (Schopenhauer), or of attentional control, as Wundt (1874) argued, which is discussed later.

**Fig. 1:**
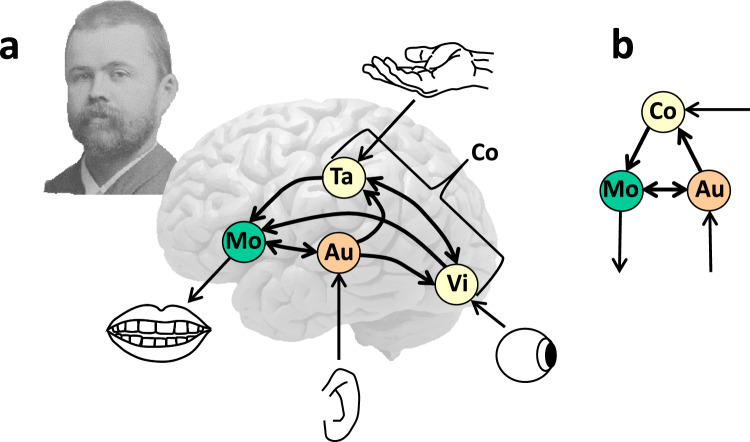
**a** Carl Wernicke (1848–1905), photographed when he began his professorship in Breslau in 1885, and an illustration of his 1874 model with associated auditory (Au) and movement (Mo) images for spoken words, which are connected to tactile (Ta), visual (Vi), and other sensory images for the concepts (Co) making up their meaning, and **b** Lichtheim’s (1885a, b) version of Wernicke’s model

Wernicke (1874) assumed that spoken word production, comprehension, and repetition proceed via associated movement, auditory, and concept images for words, as illustrated in Fig. 1a. His model diagrams were displayed on the right hemisphere in 1874, but on the left hemisphere in Wernicke (1880) and in a reprint of his 1874 monograph in Wernicke (1893). The figure illustrates the 1874/1880 version of Wernicke’s model (later modifications of the model are discussed below). In repeating a heard word, the auditory image (Au) of the word activates its movement image (Mo); in naming a seen object, the visual image (Vi) activates associated images from other modalities, including a tactile image (Ta), together making up a concept (Co), and the sensory images activate the movement image for the word; and in comprehending a heard word, the auditory image of the word activates the sensory images making up the corresponding concept. Lichtheim (1885a, b) published his own graphical version of the model, known as “Lichtheim’s house”, illustrated in Fig. 1b. Wernicke made specific assumptions about the location of the auditory and movement images for words in the brain:

The whole gyral region encircling the Sylvian fissure, together with the insular cortex, serves as the center of speech; namely, the inferior frontal gyrus, because it is motor, is the center of the movement images, the superior temporal gyrus, because it is sensory, is the center for the sound images; the fibrae propriae [association fibers] converging into the insular cortex make up the mediating psychological reflex arc. (Wernicke 1874, pp. 18–19)

Wernicke (1874) took the connection between STG and IFG via the insula, enabling repetition, to be of “immense importance for the development of language, because on it the child learns to speak. … The main task of the child learning to speak is to imitate the heard word” (p. 20). This establishes associations between auditory images of words and corresponding movement images. Next, the child learns to connect the auditory and movement images of the word with a specific concept, which consists of associated sensory images: “The concept of a bell, for example, consists of the interconnected (associated) memory images of visual, tactile, and auditory perceptions” (p. 36). After learning the associations, the sensory images that make up the concept are used to activate the corresponding movement image in naming or in spontaneous speech, although it was assumed that activation of the movement image by its auditory image remained necessary for correction (Wernicke’s 1886 proposal on how the auditory image is activated during production is explained later). Word production involves the fiber tracts “that connect the frontal lobes to the occipito-temporal lobes in the white matter of the hemisphere, especially Burdach’s arched bundle” (p. 34), referring to the AF (e.g., Catani and Mesulam 2008; Dejerine 1895; Vavassori et al. 2023). In comprehending the spoken word, its auditory image in the STG activates the associated sensory images making up the concept.

The proposed theory of aphasia is able to summarize the very different clinical pictures. This diversity itself, which hitherto gave every new observer new riddles to solve, will no longer be noticed; it can even be computed according to the laws of combination. But what is characteristic of all of them is that they are based on an interruption of the psychological reflex arc used in normal speech processes. (Wernicke 1874, p. 69)

### The patients with autopsy

Wernicke (1874) started part III of his monograph by stating that the existing aphasia literature was of little use for evaluating his new theory, because published case studies failed to report all clinical symptoms, lacked enough information about relevant aspects, or came along with incorrectly performed brain dissections. Therefore, he said to be forced to provide a different kind of evidence, coming from cases examined by himself.

The whole variety of clinical pictures of aphasia moves between two extremes, the purely motor aphasia and the purely sensory. The existence of these two forms should be regarded as irrefutable proof that two anatomically distinct centers for language exist. While pure motor aphasia is frequently found in the literature, so that there can no longer be any doubt as to its occurrence and the damage to the inferior frontal gyrus, as far as I know, not a single concise case of the pure sensory form has been reported in the literature. (p. 39)

Wernicke’s (1874) most famous patient was Susanne Rother (Case 2), although not known by name in the literature but as the first patient providing evidence for the anatomical locus of sensory aphasia. Rother was 75 years old and a stroke victim. She was dementing and presented with impaired word comprehension and fluent production with paraphasias (“confusion, complicated with aphasia”, p. 44). Wernicke had seen her in October and November 1873 (she died in December) but could not describe her language impairment in much detail since, he wrote, “my notes do not have the desired detail and accuracy, because at that time I still lacked a correct analysis of the symptom complex of aphasia” (p. 43). Given that he did not make this remark about the other patients (seen from December 1873 onward), the “correct analysis” must have dawned upon him at the end of 1873. Autopsy by Wernicke on the brain of Rother revealed damage to the left STG and generalized atrophy of the brain. He classified her as a case of *sensory* aphasia. Disruption of the auditory word images explains the word comprehension impairment and the paraphasias, as the auditory images no longer sufficiently constrain the selection of movement images.

The second patient with autopsy was Rosina Peter (Case 5), 78 years old, who suffered from strokes. She presented with severely impaired production (i.e., initially she produced “a dull, unintelligible mumble”, p. 55, and later was completely speechless) and spared comprehension. Wernicke’s autopsy revealed considerable damage to the white matter under Broca’s area and the central gyrus. He classified her as a case of *motoric* aphasia.

The third patient with autopsy was Withold von Salmonsky (Case 10), only 20 years old, who was epileptic. Wernicke saw him in March 1874, presenting with largely preserved comprehension and moderately impaired production. The patient died only two months later in May 1874. Autopsy revealed a large abscess in the left temporal lobe, while the IFG was spared. Wernicke took him to be an unclear case.

The fourth patient with autopsy (Case 8), whose dissection findings were reported in the addendum, was Louise Funke, 59 years old. She had suffered from a stroke and presented with severely impaired comprehension and severely impaired production (she only said “ja”). The autopsy revealed generalized atrophy and damage to the left IFG, the insula, and almost the whole left temporal lobe, including the STG. Wernicke ended with a rhetorical question:

If we compare this finding with that of Rother (Case 2), we see agreement because in both cases the first temporal convolution and its connectivity with the second is affected. Both suffered from sensory aphasia. Could this coincidence be due to chance? (p. 72)

### Further clarification of the model in 1886

During the mid-1880s, in a series of articles in the journal *Fortschritte der Medizin* (Progress in medicine), Wernicke critically discussed then-recent works on aphasia published by others, interwoven with his own views. In one of the articles (Wernicke 1886), he critically discussed Lichtheim’s (1885a, b) terminology for referring to the aphasia syndromes. Wernicke’s proposal for the names of seven syndromes is still in use today (e.g., Kemmerer 2022). The *cortical sensory*, *subcortical sensory*, and *transcortical sensory* aphasias were assumed to result from disruption of, respectively, the auditory images (Au in Fig. 1), the acoustic input (ear → Au), and the connections between the auditory and concept images (Au → Co) in the 1874 model. The *cortical motor*, *subcortical motor*, and *transcortical motor* aphasias result from disruption of, respectively, the movement images (Mo), their output (i.e., Mo → mouth), and the connections between concept images and the movement images (Co → Mo). Finally, *conduction* aphasia results from disrupted connections between the auditory and movement images (Au → Mo).

Wernicke (1874) had explained paraphasias in cortical sensory aphasia by assuming that the auditory image of a word provides a correction (“Correctur”, p. 23) on the selection of the appropriate movement image in naming and spontaneous speech. However, how exactly the auditory image of a word became activated had remained unclear, although reverberation of activation between movement and auditory images seemed a likely possibility (e.g., Eggert 1977). This was also Lichtheim’s (1885a, b) interpretation of Wernicke’s model (“a branch current [from Mo] to A[u]”, p. 339). In clarifying the issue, Wernicke (1886) proposed a double pathway (“auf doppeltem Wege”, p. 373): In concept-driven word production, a concept activates the corresponding movement image directly (i.e., Co → Mo) as well as indirectly via the auditory image (i.e., Co → Au → Mo). With damage to the STG, disrupting the auditory images, selection of motor images is insufficiently constrained, explaining the paraphasias in sensory aphasia. Hickok (2012) advanced a modern version of the idea of a double pathway in speech production.

More generally, Wernicke’s idea of ​​a sensory-based corrective function can be seen as a precursor to feedback control, which is now widely accepted as a core mechanism in motor planning, including speech (Guenther 2016). Hickok argued that feedback control occurs not only at low-level motor planning, but also at phonological (Hickok 2012; Walker and Hickok 2016) and syntactic (Matchin and Hickok 2020) levels. This modern work provides a principled computational explanation of Wernicke’s corrective function.

### The aphasia symptom complex of 1906

Almost two decades later, Wernicke prepared a new text on aphasia and his model, which turned out to be the final update, posthumously published as Chapter 13 in the periodical *Die deutsche Klinik am Eingange des zwanzigsten Jahrhunderts* (The German clinic at the beginning of the twentieth century). After outlining his model, Wernicke (1906) once again stressed the importance of the insula and its white matter for language:

The universally recognized importance of the insula for the language function suggests that it forms the meeting point of the paths of association through which we have to think the two centers of spoken language are linked ... Between the lentiform nucleus and the insular cortex there are two layers of white matter, … the external capsule and extreme capsule. (pp. 544–545)

In addition to the insular pathway, Wernicke (1906) also described other anatomical connections between temporal and frontal cortex in more detail, with reference to Dejerine (1895) and von Monakow (1897). He wrote:

Besides the insular cortex … two powerful association bundles come into consideration for the anatomy of the language region. … While the uncinate fasciculus exclusively connects the facing parts of the frontal lobe and temporal lobe in the shortest way, the second bundle, the arcuate bundle or arcuate fasciculus, is not really a special bundle, but the general system of sagittal directed association fibers of the convex surface of the brain. (p. 546)

Dejerine (1895) had taken the AF (“le faisceau arqué”) to terminate “in the posterior segment of the first temporal convolution (T_1_) and in the second temporal convolution (T_2_)” (p. 756). Whereas Wernicke (1874) had suggested that the AF connects temporal areas outside the STG to the IFG, he now maintained that the AF also consists of fibers commencing in the posterior third of the STG, and moreover, of fibers from the supramarginal gyrus in inferior parietal cortex. After describing how one should make the AF visible during dissection, Wernicke (1906) wrote:

One can then see that a special bundle in the deepest white matter of the marginal gyrus bends after the superior temporal gyrus and curves around the posterior extension of the Sylvian fissure. ... fibers originating from the marginal gyrus and the posterior third of the superior temporal gyrus undoubtedly also join the arcuate bundle. (p. 546)

Thus, whereas three decades earlier, Wernicke had assumed that the auditory image of a word in left STG is associated with its movement image in left IFG via the insula, he now also acknowledged involvement of the AF. Modern research distinguishes between a dorsal pathway for language, involving the AF, and a ventral pathway, involving the uncinate fasciculus (UF) and more posterior fiber tracts running through the extreme capsule, hereafter referred to as posterior EmC tracts. Weiller et al. (2021) provided evidence that the UF is the anterior, hook shaped (“uncinate”) part of a continuum of ventral tracts that pass through the extreme capsule.

## Wundt’s (1902) critique of Wernicke’s model

While Marie (1906) and Head (1926) rejected Wernicke’s model wholly, Wilhelm Wundt questioned the role of auditory images in word production and maintained that psychological reflex arcs are not sufficient to explain lexical performance and its aphasic presentation (Wundt 1874, 1880, 1896, 1902). I discuss these two issues in turn.

**Fig. 2:**
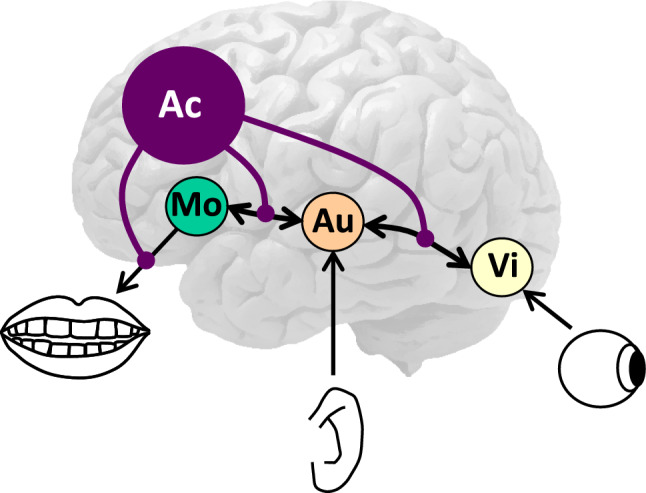
Illustration of Wundt’s (1902) proposal that conceptual elements like visual representations (Vi) are mapped onto movement representations (Mo) via auditory representations (Au) of words. To achieve selective processing (e.g., to comprehend and respond instead of repeating a word), Wundt proposed that irrelevant associations are inhibited by an attentional control (Ac) process located in the frontal lobes

A first issue raised by Wundt (1902) concerned the assumption of Wernicke (1874) that spontaneous speech is achieved by directly mapping sensory images for concepts onto movement images. He argued instead that the auditory and movement aspects of words are intimately related and therefore concepts are not directly mapped onto movement representations but only indirectly via the auditory representations, as illustrated in Fig. 2. Kussmaul (1877) and Freud (1891) had expressed the same view. Wernicke (1886) had assumed only a partial mapping via auditory images as part of his proposal of a double pathway (i.e., Co → Mo and Co → Au → Mo), and von Monakow (1897) proposed the same, stating that concepts activate in parallel “the phonetic chords (word roots and syllables) in F_3_” and “the chords for the word sounds in T_1_” (p. 509).

If concepts are mapped exclusively via auditory images onto the movement images (Freud 1891; Kussmaul 1877), and the auditory-to-movement mapping is disrupted, repetition and production impairments should go together, as empirically observed. As Wundt (1902) stated: “Transfer of what is heard into the articulated word and spontaneous articulation must then always be disturbed at the same time” (p. 313). However, Lichtheim (1885a, b) had already made clear that also with a direct mapping of concepts onto movement images, word repetition and production impairments should go together. When the mapping of auditory onto movement images is disrupted, words will be repeated via their concepts following the production route. Then, in both repetition and production, the auditory images will no longer constrain the selection of the movement image, leading to paraphasias in both. Wernicke (1906) took as proof for the intactness of the pathway mapping auditory images onto movement images “the ability to repeat, also without understanding, words or word sequences that cannot be linked with any meaning” (p. 499), nowadays referred to as the repetition of *pseudowords* (e.g., Janssen et al. 2023; Shallice and Warrington 1977).

A second issue raised by Wundt concerned the sufficiency of psychological reflex arcs to explain lexical performance and its aphasic presentation. Conceiving of mental processing in terms of psychological reflexes was widespread at the time Wernicke wrote his 1874 book (see Levelt 2013 for discussion). However, there were also dissenting voices supplementing the psychological reflexes by other mechanisms, as Wundt (1874) did in his epoch-making *Grundzüge der physiologischen Psychologie* (Principles of physiological psychology). In discussing the Wernicke-Lichtheim model, Wundt (1902) argued that its reflex-like mapping of sensory onto movement images fails to acknowledge acts of attentional control achieving selective processing, to which he referred to as “Apperception”, located in prefrontal cortex (Wundt 1880, 1896).^3^ For example, attentional control would explain why a patient named Seidel responded appropriately to a question such as “Is your name Seidel?” by saying “Yes” (taken from Wernicke 1874, p. 61). When hearing the question, the movement images of the words in it are activated, including that of “Seidel”. Why, then, did the patient not repeat the question or part of it, like “Seidel”, as patients with transcortical sensory or motor aphasia often do (Kemmerer 2022). According to Wundt, this is because the “laws of association, too, are entirely subject to the control of attention” (1874, p. 793), which “expresses itself not only in the elicitation of certain movements, but also in the perception of sense impressions and the reproduction of ideas” (p. 830). In the first four editions of the *Grundzüge* (which appeared between 1874 and 1893), Wundt conceived of attentional control as being excitatory, and in the last two editions (which appeared between 1902 and 1911) as inhibitory, with the control being optionally applied to perceptions, movements, or both (see Roelofs 2021a for discussion). Figure 2 illustrates the inhibitory version. In the example with a patient answering a question, selective responding is achieved by inhibiting the connection between the auditory and movement images, or between the movement image and the articulatory organs, for the inappropriate response “Seidel” and other words so that the appropriate response “Yes” can be produced. Modern functional neuroimaging has confirmed Wundt’s assumption that attentional control is underpinned by the frontal lobes (see Posner and Raichle 1994 for a review of the early evidence, and Badre 2020 and Posner 2012 for recent studies). Pathological repetition or echolalia is associated with damage to the medial frontal cortex (Berthier et al. 2017).

## Geschwind’s (1974) revival of Wernicke’s model

**Fig. 3:**
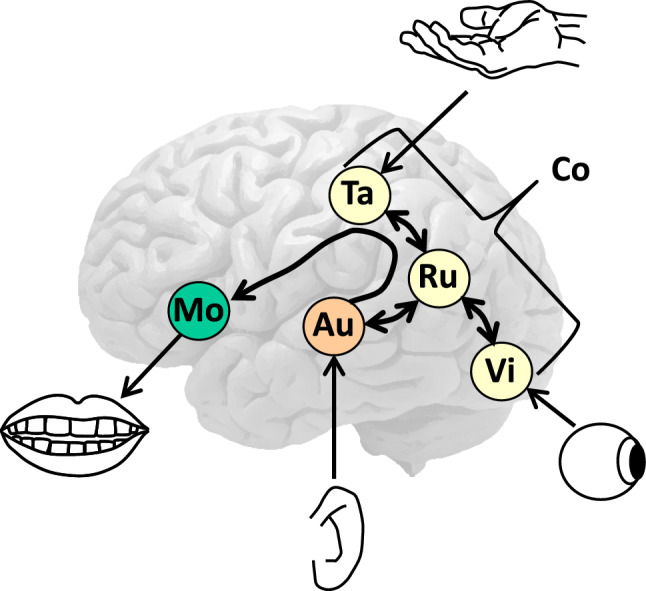
Illustration of Geschwind’s (1974) proposal that tactile (Ta), visual (Vi), and other sensory features of concepts (Co) are associated via the angular gyrus with the auditory forms (Au) of words. The angular gyrus contains the rules (Ru) linking the sensory features of concepts to auditory forms of words, which activate corresponding articulatory movement forms (Mo) via the AF

One century after it was proposed, Norman Geschwind revived Wernicke’s model in several articles (e.g., Geschwind 1970, 1972). A compilation of the articles can be found in Geschwind (1974). In his version of Wernicke’s model, Geschwind proposed that the STG subtract of the AF, directly connecting the STG and IFG, underpins both repetition and concept-driven word production. Figure 3 illustrates the model.

With reference to the English translation of Wernicke (1906) that appeared in 1908, Geschwind stated:

Wernicke (1874) hypothesized that Broca’s area and the posterior temporal auditory association area were connected by a pathway running through the insula, a lesion of which would give rise to a distinctive syndrome, *Leitungsaphasie* (conduction aphasia). Later Wernicke (1908) appeared to agree with von Monakow that the arcuate fasciculus, which runs through the parietal operculum, was a major link between the two areas. (Geschwind 1974, pp. 509–510)

Geschwind assigned a crucial role to the angular gyrus in mapping between concepts and auditory forms of words in production and comprehension (see Geschwind 1965 for extensive discussion).

Saying the name of a seen object, according to Wernicke’s model, involves the transfer of the visual pattern to the angular gyrus, which contains the “rules” for arousing the auditory form of the pattern in Wernicke’s area. From here the auditory form is transmitted by way of the arcuate fasciculus to Broca’s area. There the articulatory form is aroused, is passed to the face area of the motor cortex, and the word is spoken. … Understanding the spoken name of an object involves the transfer of the auditory stimuli from Heschl’s gyrus (the primary auditory cortex) to Wernicke’s area and then to the angular gyrus, which arouses the comparable visual pattern in the visual association cortex. (Geschwind 1972, pp. 117–118)

Geschwind referred to this account as “Wernicke’s model”, but the assumption that naming proceeds exclusively via the auditory images of words was made by Kussmaul (1877) and Wundt (1902), among others, but not by Wernicke (1874, 1906). Moreover, in support of his assumption of a cross-modal association role for the angular gyrus, Geschwind (1965) referred to Dejerine, who had assumed that this area is crucial for reading. However, Wernicke (1906) had rejected this “erroneous position of Déjérine” (p. 548) and assumed instead direct connections between occipital cortex and the perisylvian language areas. Damage to “the white matter of the lower parietal lobe offers the opportunity, in a relatively small space, to break through all connections between the centers of spoken language and the two occipital lobes” (p. 548).

Geschwind’s (1972, 1974) assumption that the AF maps auditory forms onto articulatory movement forms in both production and repetition explains why repetition and production impairments are correlated. However, explaining dissociations provides a challenge. For example, in the modern literature, Selnes et al. (2002) reported a patient with extensive damage to the AF, who presented with impaired spontaneous speech and naming but relatively spared repetition. However, if the AF lesion impairs production, it should impair repetition too. Later, I discuss evidence for double dissociation.

## Subtracts of the AF with different functions

Modern evidence supports the view that the AF underpins both concept-driven production and repetition. But different from what Geschwind (1970, 1972, 1974) assumed, production and repetition appear not to be achieved via shared fibers.

Postmortem dissection and tractography indicate that the AF includes two distinct subtracts that directly run from temporal to frontal cortex, one running from the posterior STG to the IFG and the other running from the posterior MTG to the IFG (e.g., Fernández-Miranda et al. 2015; Yagmurlu et al. 2016), as Dejerine (1895) maintained. Moreover, other *indirect* AF subtracts run from temporal to inferior parietal cortex and from parietal cortex to the IFG (e.g., Catani et al. 2005; Catani and Mesulam 2008), as Wernicke (1906) observed. Furthermore, AF fibers originate from inferior temporal gyrus, as Wernicke (1874) assumed, and terminate in premotor cortex and middle frontal gyrus (Fernández-Miranda et al. 2015; Yagmurlu et al. 2016).

**Fig. 4:**
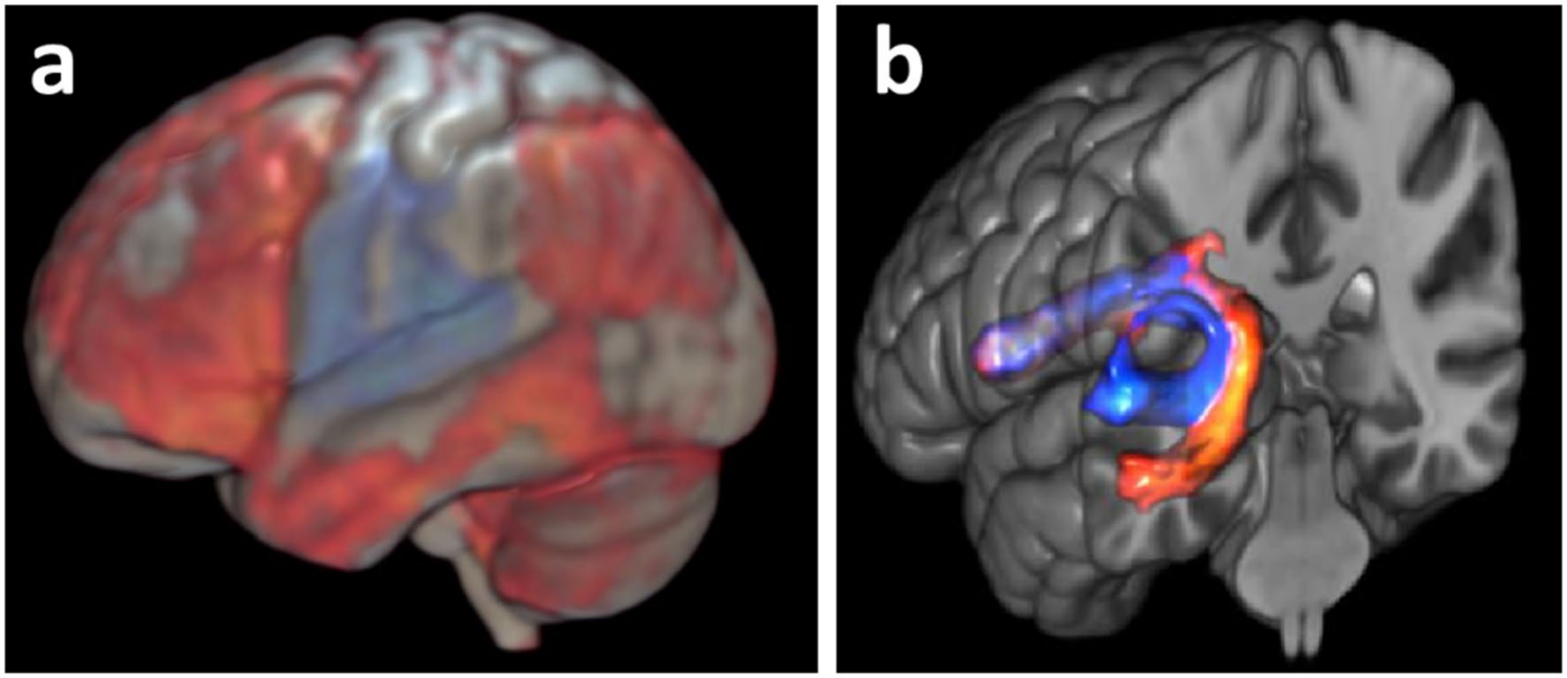
**a** Functional activation for pseudoword repetition > verb generation (blue) and verb generation > pseudoword repetition (red), and **b** left AF subtracts subserving repetition (blue) and verb generation (red) averaged across 50 participants. Adapted from Janssen et al. (2023)

Evidence from functional brain imaging and tractography indicates that the AF subtract directly running from the STG to the IFG mediates repetition and that the subtract directly running from the MTG to the IFG mediates concept-driven word production. Using deterministic tractography, Glasser and Rilling (2008) reported evidence that the STG terminations of the AF overlapped with phonological activations in prior functional neuroimaging studies and the MTG terminations overlapped with lexical-semantic activations. Combining functional magnetic resonance imaging (fMRI) with diffusion-weighted imaging and probabilistic tractography, Janssen et al. (2023) obtained evidence that the STG subtract underpins pseudoword repetition and the MTG subtract underlies verb generation tapping concept-driven word production. In the verb generation task, participants produce a verb that is appropriate to a presented noun, for example, they say “eat” in response to the heard word “apple” (e.g., Posner and Raichle 1994). Figure 4 shows the functional activations for pseudoword repetition and verb generation, and the two distinct left AF subtracts subserving these tasks. Both direct subtracts terminate in the IFG, both in the pars opercularis and in the pars triangularis.

## The WEAVER++/ARC model

Whereas the models of Wernicke (1874) and Geschwind (1974) were verbally described and displayed as diagrams, computational modeling is an important new modern advance. A computational model is a computer program that implements theoretical assumptions and can be used in simulations to see if the theory matches empirical observations and to derive new predictions that can be tested. A neurocognitive computational model is concerned with cognitive functioning and associated structures and processes of the brain (e.g., Kriegeskorte and Douglas 2018). Compared to diagrams, computational models allow for more rigorous tests of whether theoretical assumptions can account for the data, with the requirement to precisely define the nature of representation and processing, and for aphasia, also the nature of the impairment (e.g., Dell et al. 2013; Walker and Hickok 2016). Neurocognitive computational models like Lichtheim 2 of Ueno et al. (2011) have elucidated impairments of word production, comprehension, and repetition in poststroke aphasia and in one variant of primary progressive aphasia (PPA) due to neurodegeneration, namely semantic dementia. The neurocognitive WEAVER++/ARC model has been applied to poststroke aphasia (Roelofs 2014, 2021b, 2023a), the three variants of PPA (Janssen et al. 2020; Roelofs 2022), and language impairment in other neurodegenerative syndromes (Roelofs 2023b, c). The model integrates behavioral psycholinguistic, functional neuroimaging, tractography, and aphasiological evidence (WEAVER++/ARC is an acronym standing for Word Encoding by Activation and VERification / Arcuate Repetition and Conversation), see Roelofs and Ferreira (2019) for a review. The model builds on the work of Wernicke (1874) and Geschwind (1974), but also addresses Wundt’s (1902) concerns about the need for attentional control, and is therefore chosen for detailed discussion in this article. Other comparable models such as Lichtheim 2 and those of Dell et al. (2013) and Walker and Hickok (2016) do not take attentional control into account.

According to the WEAVER++/ARC model, in word production, comprehension, and repetition, three major memory systems of the human brain interact, namely declarative, procedural, and working memory. These memory systems came to be distinguished during the 1970s. When a goal (e.g., to name a picture, to point to the picture corresponding to a heard word, to repeat a word or pseudoword) is specified in working memory, information about concepts and words needed to achieve the goal is retrieved by spreading activation through an associative network stored in long-term declarative memory. Selection of relevant information is achieved by the application of condition-action rules stored in long-term procedural memory. The rules also exercise top-down attentional control. As indicated, production rules are somewhat reminiscent of Wundt’s (1880, 1896) motivated internal actions. Declarative memory is thought to be underpinned by temporal and inferior frontal regions, procedural memory by frontal regions, basal ganglia, thalamus, and cerebellum, and working memory by dorsolateral prefrontal cortex (e.g., Eichenbaum 2012 for a review).

**Fig. 5:**
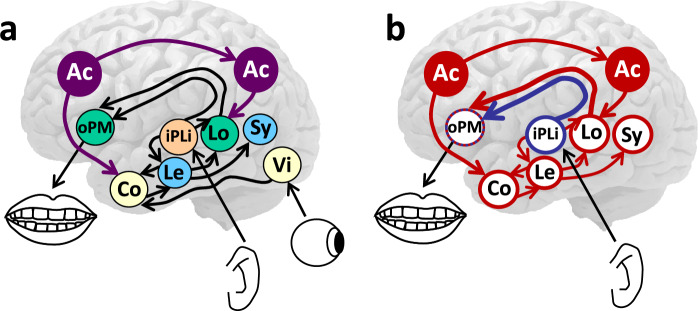
Functional neuroanatomy of the WEAVER++/ARC model (Roelofs 2014, 2022) **a** A declarative network connects visual input (Vi), concept (Co), lemma (Le), syntax (Sy), lexical output form (Lo), output phoneme and motor (oPM), and input phoneme and lexical input form (iPLi) representations. Attentional control (Ac) is achieved by a frontoparietal system operating on, for example, concepts and lexical output forms. **b** Differential deployment of processing pathways for auditory pseudoword repetition (blue) and verb generation (red)

The functional neuroanatomy assumed by WEAVER++/ARC is illustrated in Fig. 5a (see Indefrey and Levelt 2004 for a meta-analysis of neuroimaging studies on word production and listening). Modality-general representations of concepts (Co) in the anterior temporal lobes (ATLs) are connected to representations of modality-specific features in widely-distributed perceptual and motor regions (e.g., Lambon Ralph et al. 2017; Patterson et al. 2007), such as the visual shape of objects (Vi) in the posterior fusiform gyrus. Concepts are connected to lemmas (Le) in the middle part of the left MTG, which specify the syntactic properties of words (Sy), such as that *apple* is a noun and *eat* a verb. Thus, different from Wernicke (1874) and Geschwind (1974), there are concept representations (i.e., Co) separate from their perceptual features (e.g., Vi) as well as lemma representations of the syntactic properties of words. The assumption of modality-general representations of concepts explains, for example, why naming difficulty in the semantic variant of PPA occurs across input modalities, including vision, touch, and audition, with circumscribed atrophy in the ATLs but not in areas representing modality-specific features. Lemmas are connected to lexical output forms (Lo) in left posterior STG and MTG, which are connected to output phoneme and motor representations (oPM) in the IFG and premotor and motor cortex. Input phoneme and lexical input form representations (iPLi) are stored in middle to posterior STG and superior temporal sulcus (STS) bilaterally (see Kemmerer 2022 for a review). The model assumes that the direct AF subtracts connect input phonemes to output phonemes (via the STG subtract) and output lexical forms to output phonemes (via the MTG subtract), creating nonlexical and lexical phonological routes, respectively.

In picture naming, activation spreads from concepts via lemmas and lexical output forms to output phonemes and motor programs; in word comprehension, activation spreads from input phonemes and lexical input forms via lemmas to concepts; and in repetition, activation spreads from input phonemes and lexical input forms to output phonemes and motor programs, both directly (i.e., from input phonemes to output phonemes) and indirectly via lemmas and lexical output forms. Other computational models (e.g., Dell et al. 2013; Nozari et al. 2010) also assume nonlexical and lexical phonological routes to motor programs, which can be disrupted separately, as in WEAVER++/ARC. Speech input is processed via a ventral pathway for comprehension and via a dorsal pathway for repetition (Hickok and Poeppel 2007). Addressing Wundt’s (1902) concern, selective processing is achieved by an attentional control system, thought to be underpinned by frontoparietal cortex, basal ganglia, thalamus, and cerebellum, applying procedural knowledge about how to perform the tasks. In addition to these frontoparietal and basal ganglia thalamocortical systems for domain-general attentional control (Badre 2020; Posner 2012), the posterior temporal cortex specifically supports semantic control that guides concept selection in context (Jefferies and Lambon Ralph 2006; Lambon Ralph et al. 2017).

Figure 5b shows the differential deployment of processing pathways for the pseudoword repetition and verb generation tasks of Janssen et al. (2023). Pseudoword repetition proceeds from input phonemes dorsally via the direct STG subtract of the AF to the corresponding output phonemes and motor programs. This predicts activation in the STG and IFG, and engagement of the STG subtract, as observed by Janssen et al. (see Fig. 4). In contrast, verb generation proceeds from input phonemes and a lexical input form ventrally to a lemma and the corresponding object concept (e.g., apple) and then via an appropriately related action concept (e.g., eat) to its lemma and lexical output form, and dorsally via the direct MTG subtract to output phonemes and a motor program, with the attentional control system steering the process. The control is needed to sequence the processes in verb generation and to prevent inadvertent repetition of the noun (as in the earlier “Seidel” example, to comprehend and respond instead of repeating a word), which would be a predominant response. This predicts activation not only in the ATL and MTG, and engagement of the MTG subtract, but also activation (for the attentional control) in frontoparietal cortex, basal ganglia, thalamus, and cerebellum, as observed by Janssen et al. (see Fig. 4), replicating seminal findings of Posner and Raichle (1994).

When the AF is directly electrically stimulated during awake brain surgery, phonological paraphasias occur in picture naming (e.g., Giampiccolo and Duffau 2022; Sarubbo et al. 2020), which seems to contradict the claim of involvement in concept-driven word production as supported by the fMRI-tractography evidence on verb generation. However, it should be noted that the contribution of the AF in mapping concepts to articulation programs in the WEAVER++/ARC model is to connect lexical output forms and output phonemes. As a consequence, disruption of AF functioning would impair phoneme retrieval and lead to phonological errors, as observed during direct electrical stimulation (see Han et al. 2016 for converging evidence from patients with AF damage). Thus, there is no discrepancy here between findings obtained with different methods (i.e., fMRI-tractography and direct electrical stimulation).

## Damage to the AF

The WEAVER++/ARC model assumes that, different from what Wernicke (1874) maintained, the AF underpins both repetition and naming, but via separate subtracts, which differs from what Geschwind (1974) assumed. Wernicke (1906) assumed an AF subtract running from STG to IFG shared between naming and repetition as well as an AF subtract from occipito-temporal cortex involved in naming. Marchina et al. (2011) conducted a study in 30 stroke patients, examining behavioral performance, including naming and repetition, and damage to the direct AF pathway, controlling for lesion volume and damage to the UF and more posterior EmC tracts. Their findings were replicated in a follow-up study by Wang et al. (2013), who tested an additional 20 patients (i.e., 50 in total) and additionally controlled for cortical damage. Whereas Marchina et al. reported AF lesion load (i.e., percentage damage), Wang et al. reported lesion volume but not percentage damage. To relate percent accuracy to percent damage, I focus on Marchina et al.’s study when evaluating the models’ predictions, but also mention analyses of Wang et al.

To quantify the strength of the statistical evidence for the presence or absence of correlations, I performed Bayesian statistical analyses and report Bayes factors (e.g., Wagenmakers et al. 2018). A Bayes factor quantifies the evidence that the data provide for one hypothesis versus another. For example, when the Bayes factor *BF*_−0_ (i.e., subscript − 0) equals 7, the data are 7 times more likely under the H_1_ that a negative correlation is present (*BF*_+0_ for a positive correlation) than under the H_0_ of no such correlation. The Bayesian analyses were performed with JASP using Cauchy priors with default parameter settings (Wagenmakers et al.). Under a standard interpretation, a *BF* of 3–10 indicates “moderate evidence”, 10–30 “strong evidence”, 30–100 “very strong evidence”, and > 100 “extreme evidence” for one hypothesis relative to the other. The data and analyses can be obtained from the Open Science Framework at https://osf.io/k3pce/

### The necessity of the direct AF pathway for naming and repetition

Marchina et al. (2011) reported that damage to the AF impairs concept-driven word production, as evident from the picture naming and conversational performance of the patients. Repetition was also examined (and reported in a Supplementary Table) but not included in the analyses. Accuracy of performance involved naming and repeating without errors. Marchina et al. and Wang et al. (2013) did not specify the nature of the errors, but it is likely that they were mainly phonological errors, as observed with direct electrical stimulation of the AF (Giampiccolo and Duffau 2022; Sarubbo et al. 2020). As indicated, WEAVER++/ARC assumes that the two direct AF subtracts implement lexical and nonlexical phonological connections for naming and repetition, and that damage to these connections gives rise to phonological errors, as in the comparable model of Dell et al. (2013). I ran new analyses to examine naming and repetition accuracy as a function of AF damage, assessing whether the AF is necessary not only for concept-driven word production but also for repetition. Partial AF correlations were corrected for lesion volume and damage to the other tracts. Wernicke’s (1874) model predicts that naming but not repetition should be affected, whereas Geschwind’s (1974) model predicts impairment of both, as does Wernicke (1906) and WEAVER++/ARC.

**Fig. 6:**
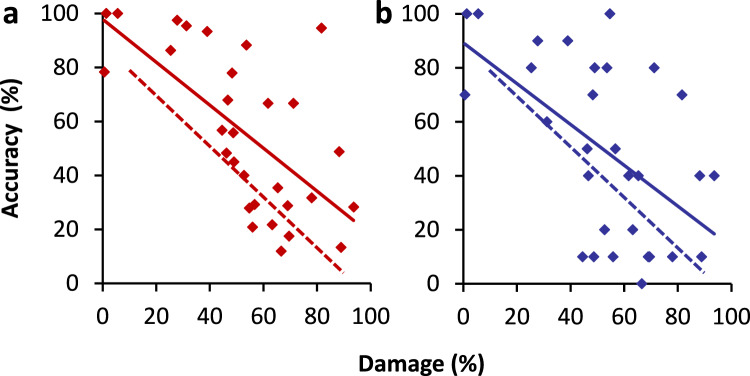
Accuracy of **a** picture naming and **b** word repetition as a function of left AF damage observed in 30 patients (diamonds and solid trendline) and in simulations with WEAVER++/ARC (dashed trendline). Patient data are from Marchina et al. (2011)

Figure 6 shows the accuracy of picture naming and word repetition as a function of left AF damage observed in the patients (diamonds and solid trendline) and in simulations with WEAVER++/ARC (dashed trendline). The figure shows that AF damage affects both naming performance (*r* =  − 0.65, *p* < 0.001, *BF*_−0_ = 590.22; partial *r* =  − 0.40, *p* < 0.039) and repetition performance (*r* =  − 0.55, *p* < 0.001, *BF*_−0_ = 56.19; partial *r* =  − 0.38, *p* < 0.051), in line with the predictions by Geschwind (1974), Wernicke (1906), and WEAVER++/ARC, but different from what Wernicke (1874) predicts.

In addition to lesion volume and damage to other tracts, it is important to rule out that the involvement of the AF is confounded with damage to cortical areas, as has been argued for repetition (Baboyan et al. 2021; Baldo et al. 2012; Rogalsky et al. 2015). Damage to the AF can lead to naming and repetition problems, but such problems do not arise exclusively from AF damage (for an extensive discussion of this in the context of WEAVER++/ARC, see Roelofs 2014, 2022, 2023b, c). Marchina et al. (2011) did not report cortical damage but Wang et al. (2013) assessed cortical damage in addition to lesion volume and damage to the other tracts. Given that they analyzed naming but not repetition, I repeated their analyses for both naming and repetition. The analyses assessed the extent to which AF lesion load affects naming and repetition compared to total lesion volume, functional gray matter lesion load, combined structural and functional lesion load, EmC lesion load, and UF lesion load. The gray matter lesion load was obtained during a speech production task performed by healthy controls. Only patients with both naming and repetition scores were included in the analyses (*N* = 45). My analyses showed that the best regression models had AF lesion load as the only predictor, with Bayes factors *BF*_10_ of 34.46 for naming and 6.65 for repetition compared to the full model with all predictors. To conclude, AF damage can cause naming and repetition deficits, taking into account lesion volume, cortical damage, and damage to other white matter tracts.

### Correlation and double dissociation

**Fig. 7:**
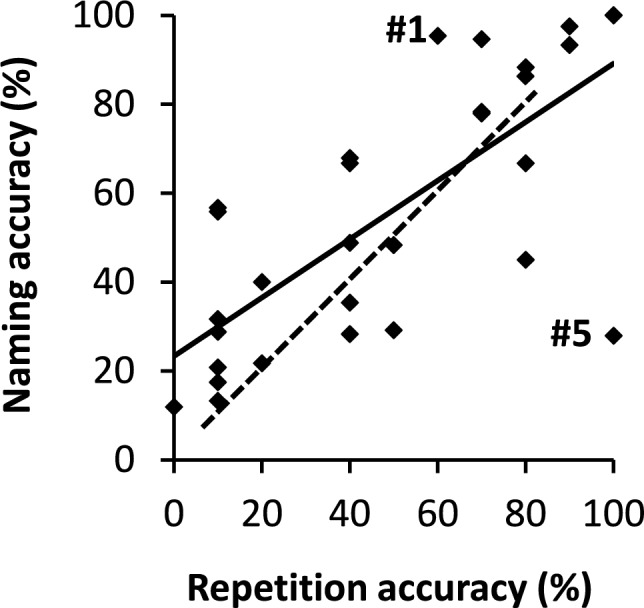
Relationship between accuracy of picture naming and word repetition observed in 30 patients with damage to the left AF (diamonds and solid trendline) and in simulations with WEAVER++/ARC (dashed trendline). Patient data are from Marchina et al. (2011). # = patient number

Figure 7 shows that the naming and repetition performances of the patients with damage to the direct AF pathway (diamonds and solid linear trendline) are positively correlated. The correlation is 0.73, *p* < 0.001, *BF*_+0_ = 10,877.77. However, the performances of a number of patients deviate from this general pattern, revealing double dissociations. Whereas patient #1 did well on picture naming (95% correct) but performed much poorer on word repetition (60% correct), patient #5 performed poorly on picture naming (28% correct) but had a perfect word repetition score (100%).

With 31% AF damage, patient #1 scored 95% correct on picture naming but only 60% correct on word repetition. The moderate repetition could be partly due to processes being impaired in repetition but not in naming, such as auditory perception. However, this would leave unexplained why picture naming remains almost unaffected with 31% AF damage. An explanation of this would require the assumption that another route is available for naming, allowing it to happen unaffected. But if such route exists, the positive correlation between repetition and naming impairment remains unexplained. Conversely, with 55% AF damage, patient #5 scored 100% correct on word repetition but only 28% correct on picture naming. Again, the poor picture naming could be partly due to processes being impaired in naming but not in repetition, such as visual perception. However, what remains unexplained is the perfect repetition performance with 55% AF damage. An explanation of this would require the assumption that another route is available for repetition, allowing it to happen unaffected. But it if such route exists, the positive correlation between repetition and naming remains unexplained.

If a single direct AF tract underlies both picture naming and word repetition, as Geschwind (1974) assumed, correlation should be observed but double dissociations are not expected with damage to the AF, contrary to the empirical observations. In contrast, the distinct AF subtract from outside the STG (for Co → Mo) assumed by Wernicke (1906) could, when specifically damaged, explain why naming may be more impaired than repetition. However, worse repetition than naming is not expected from Wernicke’s model, because the AF subtract from STG (for Au → Mo) is shared between naming and repetition. When this subtract is damaged, repetition can be achieved via concepts (i.e., Au → Co → Mo), as noted by Lichtheim (1885a, 1885b), which should yield equivalent performances for repetition and naming. The distinct direct AF subtracts for naming and repetition assumed by WEAVER++/ARC explain the observed correlation and double dissociation. Figure 7 (dashed trendline) also shows the relationship between repetition and naming accuracy in WEAVER++/ARC simulations. Repetition and naming performance are positively correlated in the model. However, repetition and naming may also double dissociate. If the 31% AF damage observed for patient #1 concerns the STG subtract, WEAVER++/ARC predicts 57% correct on word repetition and 100% correct on picture naming, which is similar to the empirically observed 60% and 95% correct, respectively. Conversely, if the 55% AF damage observed for patient #5 concerns the MTG subtract, WEAVER++/ARC predicts 34% correct on naming and 100% correct on repetition, which is similar to the empirically observed 28% and 100% correct, respectively. Marchina et al. (2011) did not distinguish the MTG and STG subtracts, thus no evidence is available on the distribution of damage across the subtracts in their patients. The anatomical proximity of the subtracts will generally cause them to be damaged together (Ivanova et al. 2021), but this does not preclude that the subtracts may be differently affected in some patients.

To conclude, when the direct AF pathway is damaged, naming and repetition performance correlate but may also double dissociate. These observations can be explained by WEAVER++/ARC, assuming different direct AF subtracts, but not by the models of Wernicke (1874, 1906) and Geschwind (1974).

### Subtracts of the AF running to and from parietal cortex

We saw that Wernicke (1906) assumed that temporal as well as parietal cortex is connected to the IFG by the AF. Modern research has confirmed that, in addition to the two AF subtracts directly connecting posterior temporal cortex to the IFG (Fernández-Miranda et al. 2015; Glasser and Rilling 2008; Janssen et al. 2023; Yagmurlu et al. 2016), the AF also includes subtracts running to and from parietal cortex (e.g., Catani et al. 2005; Catani and Mesulam 2008). A posterior subtract connects posterior temporal cortex to inferior parietal cortex and an anterior subtract connects inferior parietal cortex to the IFG. Catani et al. referred to inferior parietal cortex as “Geschwind’s territory” (p. 11), stating that “the indirect pathway appears to relate to semantically based language functions (such as auditory comprehension and vocalization of semantic content), whereas the direct pathway relates to phonologically based language functions (such as automatic repetition)” (p. 13). In contrast, the Lichtheim 2 model of Ueno et al. (2011) assumes that the indirect pathway via parietal cortex primarily underpins repetition.

Support for the assumption that the indirect AF pathway via parietal cortex underpins repetition has been provided by Forkel et al. (2020). In patients with PPA, they observed that atrophy of the posterior AF subtract (running from temporal to parietal cortex) correlated with impaired repetition performance, whereas there were no correlations with atrophy of the anterior subtract (running from parietal to frontal cortex) and the direct subtracts (running from temporal to frontal cortex). The volume of the posterior subtract was smaller in the patients than in healthy controls, while the other subtracts showed no difference between groups. On the basis of these findings, Forkel et al. concluded that the indirect rather than the direct AF pathway underpins repetition. However, this conclusion is not supported by the evidence from Janssen et al. (2023) discussed earlier (Fig. 4) and evidence from Saur et al. (2008) and Kümmerer et al. (2013). In a combined fMRI-tractography study, Saur et al. observed that areas in left STG and IFG were more active in the repetition of pseudowords than the repetition of words, and these areas were directly connected by the AF. Correspondingly, in a study of 100 aphasic stroke patients, Kümmerer et al. observed that lesion volume of this direct pathway correlated with repetition performance.

It seems that a distinction between types of repetition made by Shallice and Warrington (1977) is relevant here and may reconcile the discrepant findings. According to them, studies of repetition tend to differ in the materials used, which are biased either toward single infrequent multisyllabic words or toward lists of unconnected short familiar words. Patients with a “reproduction” impairment perform poorly on the single items but do well on the lists, whereas patients with a “short-term memory” (STM) impairment do well on the single items but poorly on the lists. Evidence indicates that inferior parietal cortex underpins the phonological store of STM (e.g., Baddeley 2003; Yue and Martin 2022) and that damage to this area impairs STM performance (e.g., Baldo and Dronkers 2006; but see Buchsbaum et al. 2011). Forkel et al. (2020) tested their patients on the repetition subtest of the Western Aphasia Battery, in which multi-word test items contribute 82% to the overall performance score. Patients with PPA typically have preserved repetition of single words and show impairment on the repetition of phrases and sentences (e.g., Lukic et al. 2019). Moreover, the indirect pathway via parietal cortex was atrophied in the patients of Forkel et al., while the direct pathway was preserved. STM impairment may explain why Forkel et al. observed that repetition performance correlated with atrophy of the posterior AF subtract. In contrast, in their studies with healthy participants, Saur et al. (2008) and Janssen et al. (2023) assessed single pseudoword repetition, which is unlikely to much engage STM but instead taxes reproduction ability, underpinned by the direct AF pathway. They obtained evidence that the direct pathway underlies repetition performance. Moreover, in their patient study, Kümmerer et al. (2013) used the repetition subtest of the Aachen Aphasia Test, in which multi-word test items only contribute 20% to the overall performance score. They observed that integrity of the direct AF pathway predicted repetition performance. Reproduction via the direct pathway explains why Saur et al., Janssen et al., and Kümmerer et al. observed that repetition performance correlated with integrity metrics of this pathway.

To conclude, the direct and indirect AF pathways contribute to repetition performance, but in different ways. Whereas the direct AF pathway underpins reproduction ability assessed by the repetition of single low-frequency words or pseudowords, the indirect AF pathway underpins STM capacity assessed by multi-word repetition, including the repetition of phrases and sentences.

## Damage to the ventral pathway

During the past century, the dorsal AF pathway has featured prominently in discussions about fiber tracts for language, whereas the ventral pathway has been somewhat neglected (e.g., Weiller et al. 2011 for a historical review and Kemmerer 2022 for an overview of the modern evidence). Still, Wernicke (1874) assumed that a ventral pathway via the insula maps auditory images onto movement images in repetition. As we saw, he assumed that two layers of white matter play a role here, the extreme capsule and the external capsule (Wernicke 1906). In the modern era, the Lichtheim 2 model of Ueno et al. (2011) assumes instead that a ventral pathway maps concepts onto motor programs. The relevant ventral tracts are taken to include the UF, which connects anterior temporal cortex to the IFG, and more posterior EmC tracts, including the inferior fronto-occipital fasciculus (IFOF), connecting the occipital lobe to the IFG (e.g., Hau et al. 2016; Sarubbo et al. 2013; Weiller et al. 2021). Ueno et al. showed that Lichtheim 2 successfully simulated impaired and spared picture naming in classic aphasia syndromes, as defined by Wernicke (1886). However, Marchina et al. (2011) and Wang et al. (2013) observed that damage to the AF, but not to the UF and posterior EmC tracts, impaired picture naming performance (for further discussion, see Hope et al. 2016 and Geller et al. 2019). These observations challenge the Lichtheim 2 model but agree with WEAVER++/ARC (Roelofs 2014).

Converging evidence for this conclusion comes from the combined fMRI-tractography study of Saur et al. (2008), who observed that areas in left STG and IFG were more active in the repetition of pseudowords than the repetition of words, and these areas were connected by the AF. In contrast, areas in MTG and IFG were more active for listening to meaningful sentences than to pseudo-sentences, and these areas were connected by fibers running through the EmC. In their study with 100 aphasic stroke patients, Kümmerer et al. (2013) observed that AF lesion volume correlated with repetition performance and EmC lesion volume with comprehension performance. One of the roles of the ventral pathway may be the attentional control of visual-semantic information processing (see the proposal of Wundt 1902, illustrated in Fig. 2). Evidence from direct electrical brain stimulation indicates that ventral tracts, including the UF, IFOF, and the inferior longitudinal fasciculus (ILF), play a causal role in visual-semantic information processing (Duffau et al. 2005; Herbet et al. 2016) and left IFG and dorsolateral prefrontal cortex in semantic control (Herbet et al. 2018). Janssen et al. (2020) tested for a role of the ventral tracts in the attentional control of picture naming by having patients with PPA name pictures in the face of distractor words, like naming a cat with the word *dog* or *xxx* superimposed. They observed that the integrity of the UF, IFOF, and ILF correlated with naming performance, lending support to the attentional control view.

## Broca’s area and beyond

In Wernicke’s model, Broca’s area plays a central role, just as in the other models discussed. However, it should be noted that in a reexamination of the brains of Broca’s historical cases, Dronkers et al. (2007) found that not only Broca’s area was damaged, but also the AF and other tracts. In a study of 134 stroke patients, Gajardo-Vidal et al. (2011) found that long-term impairments in speech production were due to damage to the white matter beneath Broca’s area, above the insula near the anterior subtract of the AF (colinear with the direct AF subtracts), without a contribution from Broca’s area itself. Fridriksson et al. (2013) also found damage to this brain region to predict fluency. Moreover, Broca’s area can be surgically removed without inducing Broca’s aphasia (Andrews et al. 2022; Duffau 2018). Still, direct electrical brain stimulation in 598 participants demonstrated speech arrest and anomia induced by stimulation of Broca’s area (Lu et al. 2021), indicating that the area does play a causal role in production. Given the abundant evidence for a role of Broca’s area in normal speech production (e.g., Flinker et al. 2015; Indefrey and Levelt 2004; Long et al. 2016; Mugler et al. 2018; Papoutsi et al. 2009; see Kemmerer 2022 for a review), spared brain regions must be able to functionally reorganize to compensate for damage to Broca’s area, perhaps including compensation by the middle precentral gyrus (Silva et al. 2022). Hickok et al. (2023) argued for a dual system of motor speech planning with a dorsal precentral area for pitch coordination and a ventral precentral area for phonetic-syllabic coordination. White matter connections, such as the fronto-striatal tract, which connects the striatum (part of the basal ganglia) and the IFG, and the frontal aslant tract, which connects the (pre)supplementary motor area and the IFG, play a role in self-initiated speech, as shown by direct electrical stimulation (Kinoshita et al. 2015).

While classical aphasia studies focused on stroke patients, in recent decades language deficits have been extensively investigated in neurodegenerative diseases (see Roelofs 2023d for a brief history and Kemmerer 2022 for a review). Regression analyses by Mesulam et al. (2021) of performance on tests of grammar, repetition, and semantics by patients with PPA (*N* = 62) revealed three non-overlapping left hemisphere clusters where atrophy was associated with reduced performance: a morphosyntactic cluster related to impaired sentence construction in the middle and inferior frontal gyri; a phono-lexical cluster related to impaired repetition in the temporoparietal junction; and a lexico-semantic cluster related to impaired picture naming and single word comprehension in the middle and anterior parts of the temporal lobe. As argued elsewhere (Roelofs 2022, 2023b, c), the assumptions of WEAVER++/ARC are consistent with these observations.

## Summary and conclusions

I have evaluated Wernicke’s (1874) central assumption of psychological reflex arcs in light of what we have learned about language in the brain during the past 150 years. According to Wernicke, repetition is mediated by a psychological reflex that is underpinned by fibers connecting left STG to the IFG via the insula, whereas fibers running from distributed posterior cortical areas to the IFG, including the AF, mediate concept-driven word production. I reviewed evidence that, different from what Wernicke initially assumed, the AF contributes to both repetition and concept-driven word production, but via separate subtracts, in line with WEAVER++/ARC but different from what Geschwind (1974) assumed. Wernicke (1906) assumed partly shared AF subtracts. In the modern WEAVER++/ARC model, the psychological reflexes are replaced by an associative network in declarative memory that is accessed by spreading activation and production rules in procedural memory selecting task-appropriate information, addressing Wundt’s (1902) concern. New analyses of patient data support the view that the AF is necessary for both repetition and concept-driven word production, and that impairments correlate but also may double dissociate. Computer simulations showed that the WEAVER++/ARC model accounts for the findings.

## Footnotes

1 All translations of the original German and French texts are by the author. The source texts may be obtained from the Open Science Framework at https://osf.io/k3pce/ or from the author.

2 Wernicke (1874) stated: “The voluntary movement is distinguished from the reflex movement by two properties, namely 1. in that it does not follow the stimulus immediately, but owes its origin to memory images of previous sensations that are only occasionally recalled by an external stimulus. Griesinger emphasized this difference and at the same time this analogy with reflex movement in his excellent treatise *On Psychological Reflex Actions* in which he traces the spontaneous movement (“psychological reflex action”) and the reflex movement back to stimuli which travel along centripetal pathways into the cerebral cortex. … 2. Spontaneous movement also differs from reflex movement in that it is rounded, distinct, adapted to the achievement of a purpose, in a word, in the already preformed form of movement; i.e., through the existing image of ​​the movement to be carried out, which, as explained above, can also be understood as a residual sensation (memory image)” (pp. 9–10).

3 Whereas Wernicke (1874) maintained that voluntary movements are psychological reflexes that have developed from real reflexes, Wundt (1880, 1896) assumed that drive actions (“Triebhandlungen”) constitute the basis for voluntary movements, which may later automatize through practice and become reflex-like (see Levelt 2013 for extensive discussion). A drive action links a motive consisting of ideational and affective aspects (i.e., a moving reason [“Beweggrund”] and a driving force [“Triebfeder”]) to an action, which may be external or internal. An act of apperception concerns a motivated action that operates internally on mental contents. The motivated actions of Wundt bear some resemblance to condition-action production rules in modern psychology (Roelofs 2021a).
